# Transcriptome profiling of the cold response and signaling pathways in *Lilium lancifolium*

**DOI:** 10.1186/1471-2164-15-203

**Published:** 2014-03-17

**Authors:** Jingmao Wang, Yang Yang, Xiaohua Liu, Jie Huang, Qing Wang, Jiahui Gu, Yingmin Lu

**Affiliations:** College of Landscape Architecture & China National Engineering Research Center for Floriculture, Beijing Forestry University, No.35 Qinghua East Road Haidian District, Beijing, 100083 China; College of soil and water conservation, Beijing Forestry University, No.35 Qinghua East Road Haidian District, Beijing, 100083 China

**Keywords:** *Lilium lancifolium*, RNA-Seq, Transcriptome, Cold response, Signaling pathway, Biochemical mechanism

## Abstract

**Background:**

*Lilium lancifolium*, a very important cold-resistant wild flower for lily cold resistance breeding, is widely distributed in southwestern and northeastern China. To gain a better understanding of the cold signaling pathway and the molecular metabolic reactions involved in the cold response, we performed a genome-wide transcriptional analysis using RNA-Seq.

**Results:**

Approximately 104,703 million clean 90- bp paired-end reads were obtained from three libraries (CK 0 h, Cold-treated 2 h and 16 h at 4°C); 18,736 unigenes showed similarity to known proteins in the Swiss-Prot protein database, and 15,898, 13,705 and 1849 unigenes aligned to existing sequences in the KEGG and COG databases (comprising 25 COG categories) and formed 12 SOM clusters, respectively. Based on qRT-PCR results, we studied three signal regulation pathways —the Ca^2+^ and ABA independent/dependent pathways —that conduct cold signals to signal transduction genes such as *LlICE* and *LlCDPK* and transcription factor genes such as *LlDREB1/CBF*, *LlAP2/EREBP*, *LlNAC1*, *LlR2R3-MYB* and *LlBZIP,* which were expressed highly in bulb. *LlFAD3*, *Llβ-amylase*, *LlP5CS* and *LlCLS* responded to cold and enhanced adaptation processes that involve changes in the expression of transcripts related to cellular osmoprotectants and carbohydrate metabolism during cold stress.

**Conclusions:**

Our study of differentially expressed genes involved in cold-related metabolic pathways and transcription factors facilitated the discovery of cold-resistance genes and the cold signal transcriptional networks, and identified potential key components in the regulation of the cold response in *L lancifolium*, which will be most beneficial for further research and in-depth exploration of cold-resistance breeding candidate genes in lily.

**Electronic supplementary material:**

The online version of this article (doi:10.1186/1471-2164-15-203) contains supplementary material, which is available to authorized users.

## Background

Plants have a remarkable ability to cope with highly variable environmental abiotic stresses, including cold, drought, heat, salinity and nutrient deficiencies. Nevertheless, these stresses together represent the primary cause of plant injuries and losses worldwide, reducing the ornamental value and production of most major plants and crops by more than 50% [1]. As a wild cold-resistant plant, *Lilium lancifolium* is mainly distributed in the North Temperate Zone, where the winter temperate can fall as low as -35°C, but it can survive exposure, acclimate to low or freezing temperatures and continuously germinate in the next spring. In addition, studies have shown its capacity for resisting heat, drought and changing soil salinity. Nevertheless, the decline of *L. lancifolium* is gradually becoming more serious in recent years with the deterioration of its ecological environment. Therefore, further protection and a better understanding of the gene expression profile of *L. lancifolium* under cold stress is imperative, and it could be an ideal model to study cold tolerance mechanisms and signaling regulation for improving the quality of cold resistance in other plants using molecular biological techniques.

Cold responses have been observed in many plants, which initiate reactions of the freezing tolerance of plants after low temperature stress, including *Arabidopsis*[2], *Oryza sativa*[3], *Triticum aestivum*[4], and *Ammopiptanthus mongolicus*[5]. The initiation of most stress treatments correlates with a cytosolic calcium release, in some cases with stimulus-specific patterns of oscillation [6]. In addition, stimulus-specific changes in gene expression are often observed alongside a set of shared stress responses. For example, in a survey of 1, 300 Arabidopsis genes, the majority of cold and drought stress regulated genes were observed in the shared stress response [7]. Together, these observations support the hypothesis that a common set of signal transduction pathways are triggered during many stress responses. Many of the biochemical, molecular and physiological changes that occur during the cold response are considered to be important in the induction of freezing tolerance. During this process, plants alter the expression of certain genes as well as the biosynthesis of amino acids and soluble sugars [8]. At the gene expression level, DNA microarray analysis studies have revealed that exposure of *Arabidopsis* plants to a low temperature of 4°C resulted in up- or down-regulation of hundreds to thousands of cold-regulated (*COR*) genes, and that many of the cold inducible genes are linked with the accumulation of osmolytes, cryoprotectants, antioxidant detoxification enzymes, chaperones, transporters, dehydrins, late embryogenesis abundant (*LEA*) proteins and enzymes involved in lipid, carbohydrate and secondary metabolites, and in abscisic acid (ABA) and jasmonic acid (JA) biosynthesis [9].

Traditional cloning and genetic transformation methods are expensive and time consuming. In recent years, the development of novel high-throughput sequencing technologies, such as Solexa/Illumina RNA-Seq (RNA sequencing) and digital gene expression (DGE) has provided an opportunity to explore cold resistance and signaling-associated genes in different species by *de novo* assembly or mapping, and also facilitated rapid identification and analysis of the vast majority of transcriptomes [10]. Transcriptome sequencing is an efficient means to generate functional genomic data for non-model organisms or those with genome characteristics prohibitive to whole-genome sequencing [11]. Illumina/Solexa has been successfully applied to the transcriptome sequencing of many plant species, including *Populus euphratica*[12], *Aegilops variabilis*[13], *Aechmea fasciata*[14], *Brassica napus*[15], *Zea mays*[16], *Arachis hypogaea*[17], and *Picrorhiza kurrooa*[18]. For instance, transcriptome profiling of grapefruit flavedo following exposure to low temperature and conditioning treatments has increased our understanding of the principal molecular components involved in chilling tolerance and susceptibility [19]. Using Illumina/Solexa, we found that the study of differentially expressed genes involved in cold-related metabolic pathways and transcription factors could facilitate the discovery of cold-resistance genes for the desert shrub *Ammopiptanthus mongolicus*[20]. The transcriptomic analysis of *Aechmea fasciata* treated with ethylene has been revealed part of the ethylene signaling pathway and flowering process [14]. Genome-wide analysis approaches have been used to elucidate gene expression in response to drought stress in *Populus simoniia*[21].

However, few studies have been carried out to date on the cold-related metabolic pathways and transcription factors in *L. lancifolium*. Such studies would bridge the physiological and anatomical changes during cold acclimation with molecular data. Here, we present three cDNA libraries (two cold-treated and a control) of living *L. lancifolium* leaves which were subjected to short-term cold (4°C) stress treatment and describe the short-term cold response (0 to 16 h) of *L. lancifolium* using the next-generation Illumina/Solexa sequencing technology, and also compared the long-term cold acclimation (1 to 20d) of *L. lancifolium* and Oriental hybrids using anatomical and physiological analyses and biochemistry experiments. We had two specific objectives. First, to identify genes that change expressions in a stress-specific fashion and reveal the transcription factors that change in the key transcriptional, finding development networks and signal pathways in the response to cold stress. Second, to identify the expression of various genes that are co-regulated during the biological processes, such as intercellular osmoprotectant biosynthesis and biodegradation of carbohydrates of shared stress responses. This global view illustrates the “fluid” nature of the transcriptome and the challenge we face in understanding the complexity of any given stress response. In particular, the analyses on differentially expressed genes under cold stress furthers our understanding of the cold response mechanism of *L. lancifolium*, and these cold-related genes should also contribute to providing a method of developing cold-tolerant plants through genetic manipulation.

## Results

### High-throughput transcriptome sequencing and read assembly

To identify the number of genes involved in the transcriptome, a cDNA sample was prepared from an equal mixture of total RNA isolated from leaves for three libraries (cold-treated 2 h, 16 h and control 0 h samples), which were sequenced using the Illumina HiSeq™ 2000. We obtained approximately 74 million raw reads for the two cold-treated samples (CT2h and CT16h) and 41 million for the control sample (CK0h). We discarded low-quality reads, which contained adapters and unknown or low-quality bases, according to our bioinformatics analysis. After stringent quality checks and data cleaning, we obtained 115,421,520 raw reads containing a total of 11.6 Gb nucleotides. The average read size, Q20 percentage (sequencing error rate < 1%), and GC (guanine + cytosine) percentage were 90 bp, 98.1%, and 43.7%, respectively. Based on the high quality reads, 46,516 contigs were assembled with an average length of 793 bp. With paired-end joining and gap-filling, the contigs were further assembled into 39,154 scaffolds with an average length of 951 bp, including 11478 scaffolds larger than 1000 bp. After local assembly with the unmapped ends to fill in the small gaps within the scaffolds, the *de novo* assembly yielded 37,843 unigenes with an average length of 971 bp (Tables 1 and 2). To demonstrate the quality of sequencing data, ten unigenes were randomly selected and ten pairs of primers were designed for qRT-PCR, and then the products were confirmed by biological Sanger sequencing.

**Table 1:** **Overview of the sequencing and assembly**

Sample ID	Raw reads (MB)	Raw bases (GB)	Q20 value (%)	Raw reads	Quality trimed	Adaptor trimed	Number Clean reads	Clean ratio
Control-0 h	36.5	3.7	98.%	36,521,060	34,375,258	33,947,498	33,259,676	91.1%
Treatment-2 h	37.1	3.7	98.1%	37,063,886	35,088,672	34,672,716	33,979,556	91.7%
Treatment-16 h	41.8	4.2	98.1%	41,836,574	38,733,184	38,229,688	37,465,870	89.6%

**Table 2:** **Summary for the** ***Lilium lancifolium*** **transcriptome**

Statistics	Counts	Total Length (bp)	N25 (bp)	N50 (bp)	N75 (bp)	Average length	Longest (bp)	N%	GC%	Annotation counts	Annotation ratio %
Contigs	46,516	36,896,235	1,706	962	582	793	14,570	0.9	43.7		
Primary uniGene	39,154	37,242,700	1,919	1,106	648	951	14,570	0.9	43.7		
Final uniGene	37,843	36,756,285	1,963	1,146	663	971	14,570	1.0	43.6	18,736	49.5

To validate and annotate the of assembled unigenes, using E-value < 1e-5, they were blast searched against the UniProt (date: 2013.04) and Swiss-Prot protein database (date: 2013.05) (http://www.expasy.ch/sprot) which has the largest and most detailed protein annotation database, including 24,889,084 proteins. The results indicated that among the 37,843 unigenes, 18,736 (49.5% of the total) had significant similarity to known proteins in the Swiss-Prot database. The lack of *L. lancifolium* genome and EST information meant that 19.107 (50.5% of the total) unigenes had no Swiss-Prot annotation (Table 2).

### Gene annotation and functional classification

**Figure 1:**
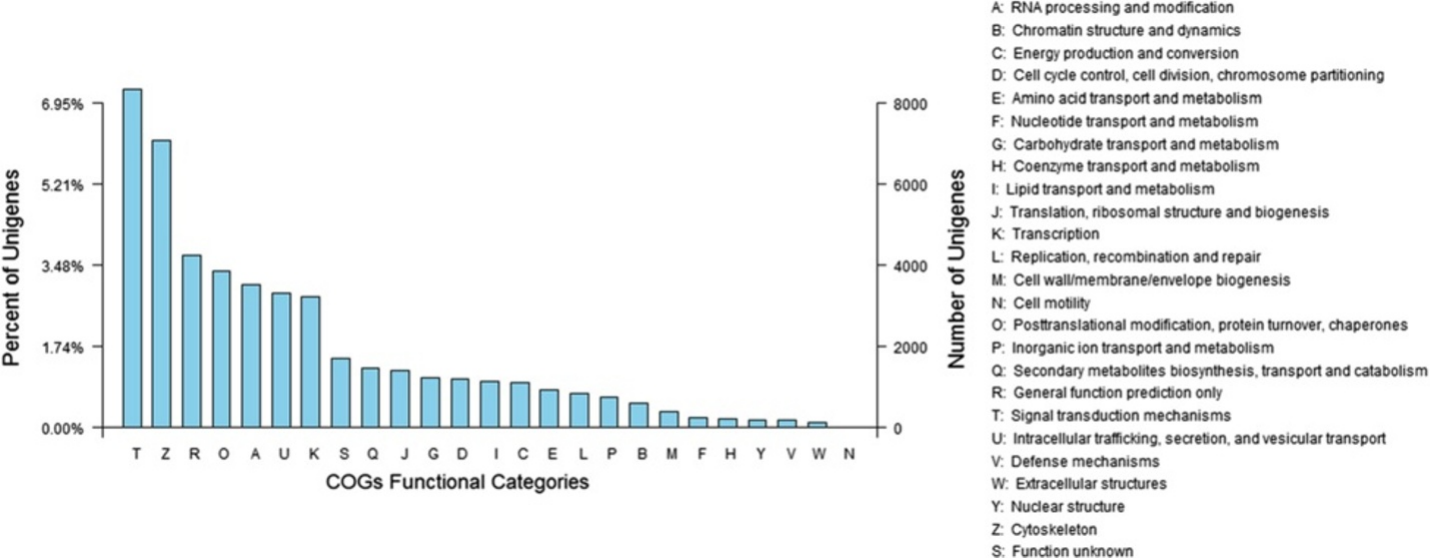
**Clusters of orthologous groups (COG) classifications in** ***Lilium lancifolium*** **.** These 13705 sequences have a COG classification within the 25 categories.

To further evaluate the completeness of our transcriptome library and the effectiveness of our annotation process, we randomly searched the annotated sequences for genes with COG (Clusters of orthologous groups) classifications. Of 18,736 Swiss-Prot hits, 13,705 sequences had a COG classification. Among the 25 COG categories, the cluster for 'Signal transduction mechanisms’ (8332, 17.54%) represented the largest group, followed by 'Cytoskeleton’ (7078, 14.89%) and 'General function prediction only’ (4239, 8.92%). The 'defense mechanisms’ (190, 0.39%) and 'extracellular structures’ (147, 0.30%) categories, represented the smallest groups (Figure 1). Compared with the control sample, the most-abundant cluster in the cold treated samples was 'Signal transduction mechanisms', indicating that STM genes play a vital regulation role in the *L. lancifolium* cold senescence and stress responses.

**Figure 2:**
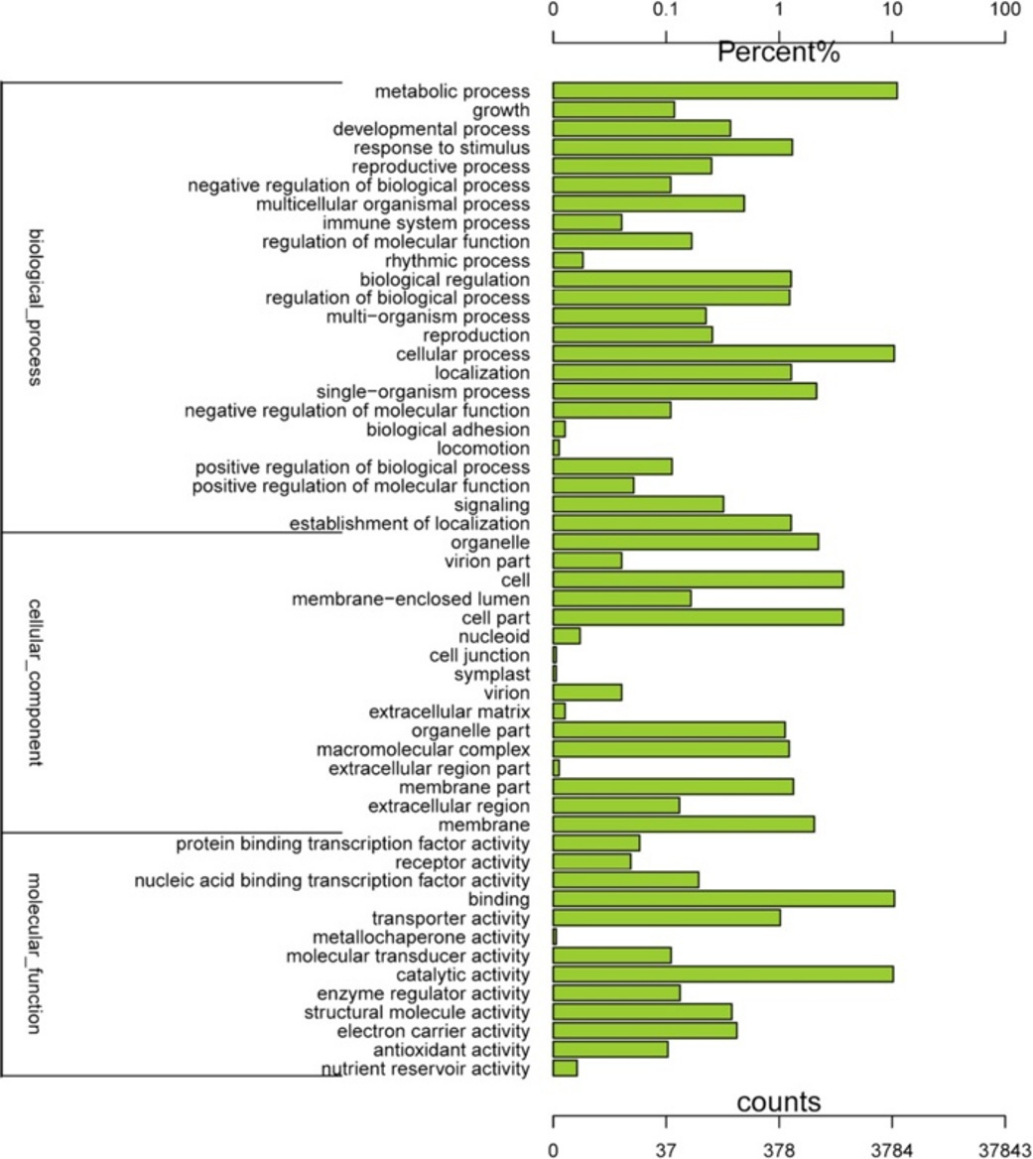
**Histogram presentation of Gene Ontology classifications.** The results are summarized in three main categories: biological process, cellular component, and molecular function. The y-axis on the right side indicates the percent of genes in a category, and the y-axis on the left side means the number of genes.

The Gene ontology (GO) assignments system was also used to classify the functions of the predicted *L. lancifolium* genes. Based on sequence homology, the 18,736 sequences were categorized into 53 functional groups (Figure 2). The unigenes were then assigned into three main categories: biological process, cellular component, and molecular function, with 7901 (42.17%), 2338 (12.47%), and 4459 (23.79%) unigenes, respectively, assigned to each. In each of the three main GO classifications, the 'metabolic process', 'cellular process', and 'catalytic activity’ terms, were dominant. We also noticed a high percentage of genes from the 'cellular process', 'cell part', and 'binding’ categories, but few from 'cell junction', 'symplast', 'extracellular region part’ and 'locomotion’ (Figure 2). The GO analysis indicated that a great number of identified genes were associated with various biological processes and molecular functions under low temperature. About 5331 sequences were annotated as belonging to the 'metabolic process’ category, which may allow for the identification of novel genes involved in secondary metabolism pathways in cold acclimation. The 'cellular component part’ suggested that the membrane and cell junction could function in the later cold resistance process (Figure 2).

The KEGG (Kyoto Encyclopedia of Genes and Genomes) is a database resource for understanding the high-level functions and utilities of biological systems, such as the cell, the organism and the ecosystem, from molecular-level information, especially large-scale molecular datasets generated by genome sequencing and other high-throughput experimental technologies (see the release notes for new and updated features). The pathway database records networks of molecular interactions in cells, and variants specific to particular organisms. Based on a comparison against the KEGG database using BLASTx with an E-value cutoff of < 10^-5^, of the 37,843 unigenes, 15,898 (42%) had significant matches in the database and were assigned to 119 KEGG pathways (Table 3). The most represented pathways were 'metabolism pathways’ (1784 members, 11.23%), 'biosynthesis of secondary metabolites’ (946 members, 5.95%), and 'Microbial metabolism in diverse environments’ (400 members, 2.51%) (Table 3). These annotations provide a valuable resource for investigating the processes, functions, and pathways involved in the short-term cold response and long-term cold acclimation.

**Table 3:** **Categorization of** ***Lilium lancifolium*** **unigenes to KEGG biochemical pathways**

KEGG categories	Mapped-ko	Unigene-NUM	Ratio of no.	Pathway-ID
Metabolic pathways	734	1784	11.23	ko01100
Biosynthesis of secondary metabolites	327	946	5.95	ko01110
Microbial metabolism in diverse environments	125	400	2.51	ko01120
mRNA surveillance pathway	48	256	1.61	ko03015
Ribosome	105	251	1.57	ko03010
Pyrimidine metabolism	63	250	1.57	ko00240
Biosynthesis of amino acids	95	237	1.49	ko01230
Cell cycle	50	233	1.46	ko04110
Carbon metabolism	71	229	1.44	ko01200
Splice some	87	223	1.40	ko03040
Protein processing in endoplasmic reticulum	71	208	1.31	ko04141
RNA transport	90	198	1.24	ko03013
Starch and sucrose metabolism	33	186	1.16	ko00500
Purine metabolism	77	169	1.06	ko00230
Plant-pathogen interaction	27	163	1.02	ko04626
Plant hormone signal transduction	38	158	0.97	ko04075
Epstein-barr virus infection	55	151	0.94	ko05169
Oxidative phosphorylation	70	150	0.94	ko00190
Homologous recombination	23	147	0.92	ko03440
RNA degradation	45	131	0.82	ko03018
Ubiquitin mediated proteolysis	55	128	0.81	ko04120
Glycolysis/Gluconeogenesis	30	128	0.81	ko00010
Endocytosis	38	127	0.79	ko04144
Amino sugar and nucleotide sugar metabolism	37	127	0.79	ko00520
Basal transcription factors	27	117	0.74	ko03022
Phenylpropanoid biosynthesis	16	109	0.68	ko00940
Ribosome biogenesis in eukaryotes	51	107	0.67	ko03008
Oocyte meiosis	29	106	0.66	ko04114
Viral carcinogenesis	38	104	0.66	ko05203
Cell cycle-yeast	47	102	0.64	ko04111
Insulin signaling pathway	19	102	0.64	ko04910
HTLV-I infection	40	101	0.63	ko05166
Fatty acid metabolism	10	100	0.62	ko00071
Pyruvate metabolism	28	96	0.60	ko00620
Others		7874	11.22	

### Changes in gene expression profiles among the different cold stress stages

**Figure 3:**
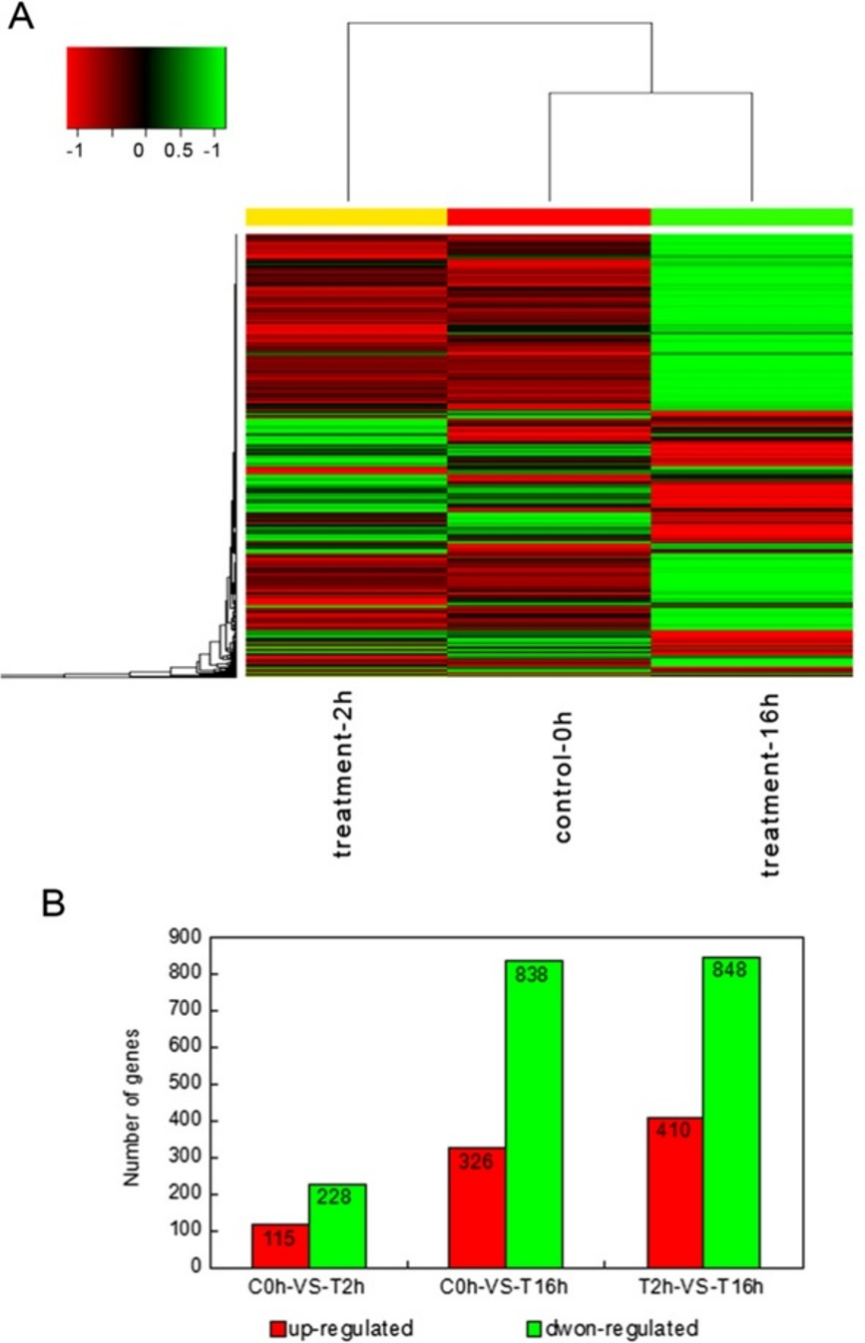
**The expression of the gene changes among the different cold stress stages. A**. The heat-map of the total differentially expressed genes (DEGs). Columns and rows in the heat maps represent samples and genes, respectively. Sample names are displayed below the heat maps. CK Results of controls, CT2h and 16h results of cold treatments. Color scale indicates fold changes of gene expression. A fold change of ≥1 is shown in green (increased transcript abundance), a fold change of ≤ -1 is shown in red (decreased transcript abundance), and no change is indicated in black. The results show that 1,028 transcripts were differentially expressed between the control and cold treatments 2 h and 16 h. **B**. Changes in gene expression profile among the different cold stress stages. The number of up-regulated and down-regulated genes between C0h-VS-T2h, C0h-VS-T16h and T2h-VS-T16h are summarized. Between the C0h and T2h *Lilium lancifolium* libraries, there are 115 genes up-regulated and 228 genes down-regulated, while there are 326 up-regulated genes and 828 down-regulated genes between the C0h and T16h *Lilium lancifolium* libraries, and 410 up-regulated genes 848 down-regulated genes between the C2h and T16h *Lilium lancifolium* libraries.

To investigate the annotated transcriptome assembly and gain statistical confirmation of the differences in gene expression that served as a reference for RNA-Seq profiling of stage-specific expression, we conducted a small RNA-Seq experiment using tangential cryosections of CK0h, CT2h and CT16h of *L. lancifolium* and mapped the resulting reads to our reference transcriptome. To minimize false positives and negatives, we concluded that a statistical analysis was reliable when applied to genes with an RPKM value ≥ 2 in at least one of the three stages. It should be noted that this statistical significance was based on expected sampling distributions. To determine which of the 37,843 genes were differentially expressed among the three stages, we filtered with an FDR ≤ 0.001 and |log_2_ (ratio)| ≥ 2; the expression of 2755 DEGs was found to be significantly changed during the three stages. On the one hand, some gene were down-regulated from the 0 h to 2 h, but up-regulated obviously at 16 h. On the other hand, some genes showed increased quantitative expression at 2 h, but decreased transcript abundance at the 16 h stage. To identify genes showing a significant change in expression during different cold stress stages, the differentially expressed tags between the three samples were identified using an algorithm developed from the heat-map. Some genes were immediately expressed at the initial stage of cold stress, while others were up-regulated subsequently indicating that transcription factors induced the expression of cold-related genes during the regulation of cold signaling (Figure 3A).

In addition, we compared the Ck0h and CT2h libraries, and 343 variable genes were found a total of 115 up-regulated and 228 down-regulated genes were detected between the two *L. lancifolium* libraries. There were also 326 up-regulated and 828 down-regulated genes between the C0h and T16h libraries, 410 up-regulated and 848 down-regulated genes between the C2h and T16h libraries (Figure 3B). This suggests that the differentiation of expressed genes between C2h and T16h is larger than that between C0h and T16h, while the difference between and the C0h and T2h is the smallest of the three. That means, in *L. lancifolium,* transcript abundance changed dramatically at these key switches among the cold stress stages of 2 h to 16 h which the cold response genes could be induced and expressed largely, but we should not ignore the genes expression during the short-term of 0 h to 2 h cold treatment, because many important cold-stress response genes were up- and down-regulated in this period, they would earliest determine the plant to play instantaneous reflection and response to the cold stress. These findings suggested forecast that our analysis was capable of identifying cold stress response genes and therefore suitable for further investigation of the biological functions of these genes.

### SOM cluster analysis of gene expression

**Figure 4:**
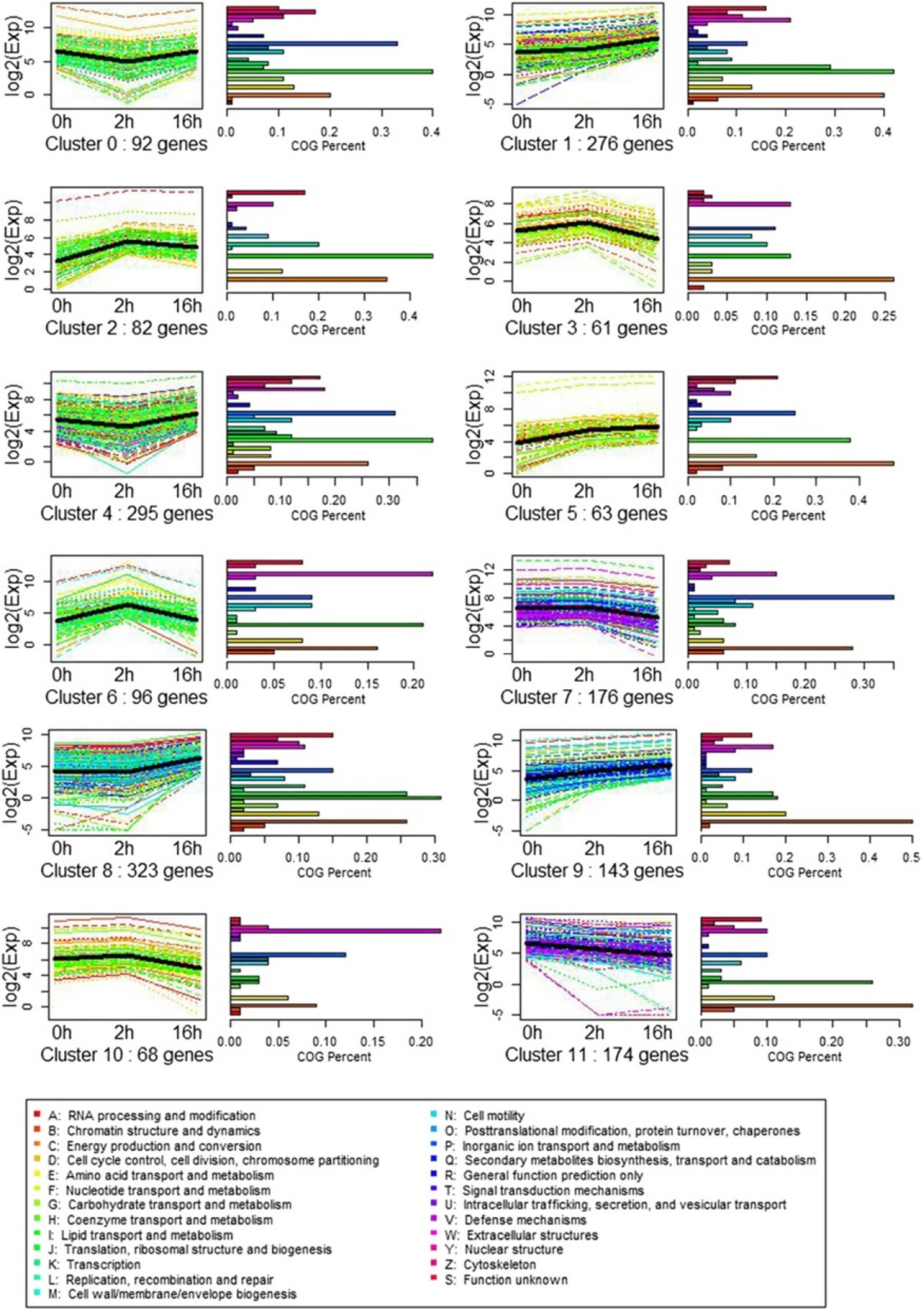
**SOM cluster analysis of gene expression in the 12 different patterns.** Clusters were obtained by the k-means method on the gene expression profiles of the 1849 modulated genes. The most abundant group is Cluster 8 and 1, with 323 and 276 genes whose expressions show positive slopes during stage of T2h to T16h embryogenesis. The second abundant group is Cluster 4, which contained 295 genes whose expression shows a negative slope from C0h to T2h.

To facilitate cluster analysis of gene expression, the expression profiles of the differentially expressed genes were determined by SOM cluster analysis based on the k-means method using Pearson’s correlation distance. The total differential genes were divided into 12 groups based on their expression modulation with analysis of GO and KEGG pathway enrichment, representing the number of profiles as indicated by figure of merit analysis. Clusters were obtained by the k-means method using the gene expression profiles of the 1849 modulated genes. The most abundant group were Clusters 8 and 1, with 323 and 276 genes whose expression showed a positive slope during the T2h to T16h stage. The second most abundant group was Cluster 4, which contained 295 genes whose expression showed a negative slope from C0h to T2h. The functional category distribution frequency was then calculated for each cluster to identify differences in the distribution of genes among the three cold stress stages (Figure 4).

Clusters 2, 3, 6, 7, and 10 comprised 483 genes up-regulation at CT2h but down-regulation in CT16h, including different pathways as the 'energy production and conversion', 'inorganic ion transport and metabolism', 'extracellular structures', 'lipid transport and metabolism’ and 'coenzyme transport and metabolism’genes. They indicate that cells accept the cold signal and instantly reflect using the ion transport and extracellular structure activities during the CT2h stage. In total, 802 genes up-regulated in clusters 1, 5, 8 and 9 in whole stages of cold response. They are specifically included in different pathways, such as 'carbohydrate transport and metabolism', 'amino acid transport and metabolism', 'translation, ribosomal structure and biogenesis’ 'energy production and conversion', and 'signal transduction and mechanism’ (Figure 4), suggesting that *L. lancifolium* initiates carbohydrate conversion and metabolism during CT2h to 16 h.

### Response of important transcription factors to cold stress

**Figure 5:**
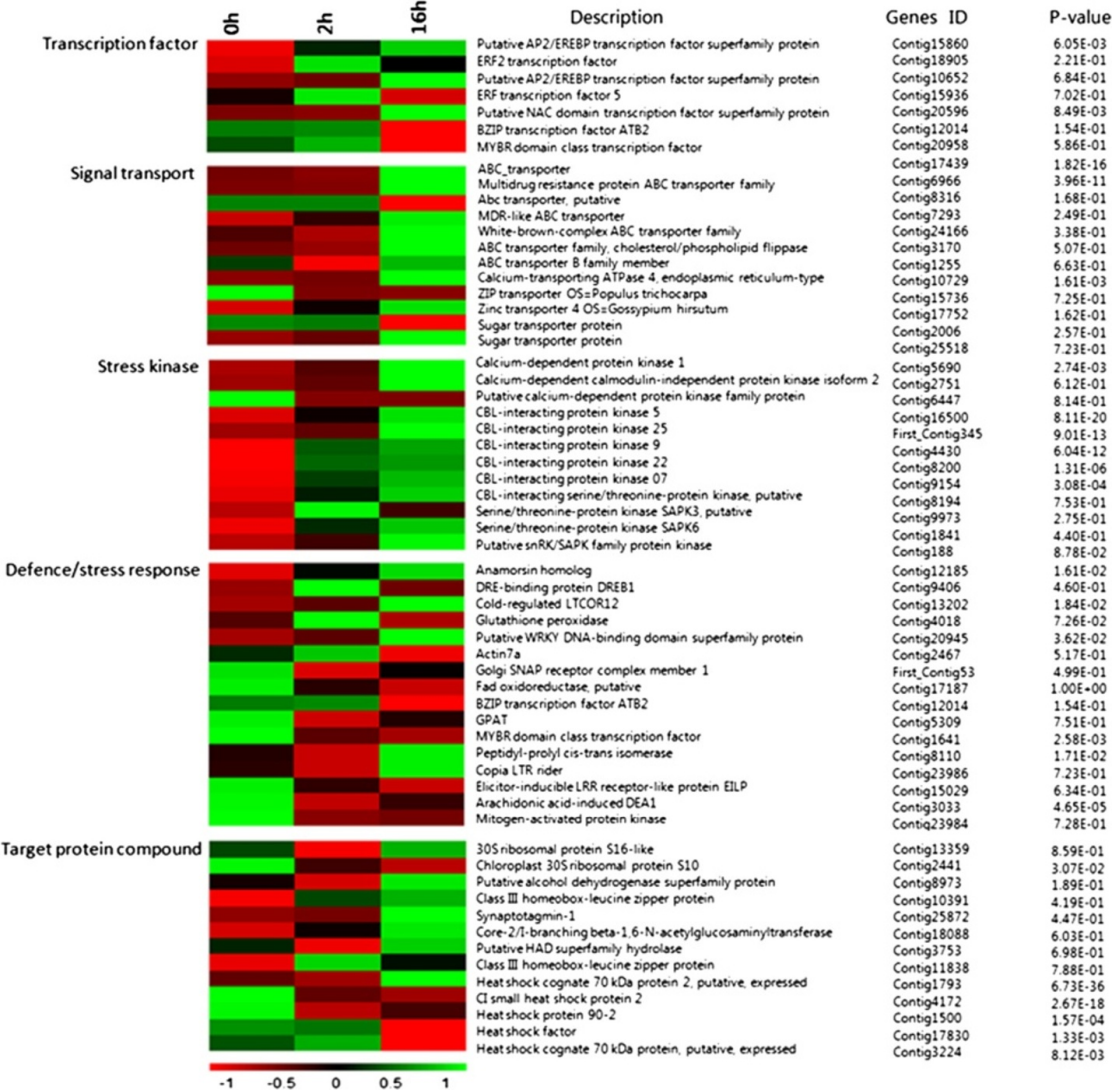
**Heat-map of 65 differentially expressed genes involved in transcription factor, signal transport, stress kinase, defense/stress response, target protein compound in the cold response and acclimation of** ***Lilium lancifolium*** **.** They were differentially expressed between the control 0 h, cold treatments 2 h and 16 h. The bar represents the scale of the expression levels for each gene (log_10_ RPKM (number of reads per kilobase per million clean reads)) in the cold response and acclimation as indicated by green/red rectangles. Green indicates up-regulation of genes and Red indicates down-regulation and no change is indicated in black. Complete information for each gene list can be found in Additional file 1: Table S1.

Transcription factors (TF) play crucial roles not only as molecular switches for gene expression, but also as terminal points of signal transduction in the response to cold stress. At the 2 h and 16 h stages cold treatment for *L. lancifolium*, the genes whose transcript abundance exhibited highly dynamic changes (|log_2_(ratio)| ≥ 4, Figure 5) included genes for transcription factors (*LlAP2/EREBP* (KJ489026) transcription protein, *LlNA*C (KJ467622) domain transcription, *LlERF2* transcription factor, *LlBZIP* transcription factor protein, *LlMYBR* (KJ467623) family domain class transcription factor), signal transport (ABC transporter, *LlCalcium*-transporting ATPase 4, *LlZIP* transporter, Zinc transporter, sugar transporter protein), stress kinases (*LlCalcium*-dependent protein kinase 1 (KJ467621), *LlCBL*-interacting protein kinase 25, Serine/threonine-protein kinase *LlSAPK3*, Putative *LlsnRK/SAPK* family protein kinase), low temperature induced-like proteins (DRE-binding protein *LlDREB1/CBF* (KJ467618), Cold-regulated *LlCOR12* (KJ489025), Putative WRKY DNA-binding domain superfamily protein, Peptidyl-prolyl cis-trans isomerase, Copia *LlLTR* rider, Elicitor-inducible *LlLRR*, Arachidonic acid-induced *LlDEA1*), and stress-associated compound proteins (Ll30S ribosomal protein, LlClass III homeobox-leucine zipper protein, LlHeat shock cognate 70 kDa protein 2 (KJ467620), *LlGPAT* protein (KJ467617), Calcium-dependent calmodulin-independent protein (KJ467621); Figure 5, Additional file 1: Table S1).

Therefore, the changes in the expression patterns of distinct transcripts suggest a requirement for different developmental events when *L. lancifolium* is under cold stress. For example, seven transcription factors preferentially expressed and 12 signal transport transcripts accumulated to a higher level at 2 h than 16 h, which indicated that transcription factors reacted more actively to cold in the initial short-term response rather than the long-term cold acclimation (Figure 5, Additional file 1: Table S1). Transcription factors (TF) act as switches and terminal points of signal transduction in a stress-specific fashion in the response to cold stress. In addition, 12 stress kinase protein genes and 13 stress-associated compound protein genes showed the highest accumulation in the cold-treatment 16 h stages which was expected, as expected for cold resistance. Interestingly, the 16 genes related to low temperature induced-like proteins showed different trends. Three of them showed higher expression in CT2h and then decreased in CT16h, such as DRE-binding protein *LlDREB1*, *LlCBL*-interacting protein kinase, Glutathione peroxidase and *LlBZIP* transcription factor ATB2, and five of them reached the highest expression value at 16 h, including Cold-regulated *LlCOR12*, *LlWRKY* DNA-binding domain superfamily protein, Copia *LlLTR* rider and Peptidyl-prolyl cis-trans isomerase; the rest of them were down-regulated from 0 h to 16 h (Figure 5, Additional file 1: Table S1). All of these results may contribute to identifying the signaling development networks in response in the cold reponse of *L. lancifolium*.

### Verification of the gene expression profiles by qRT-PCR

To further verify the expression profiles of the genes in our Illumina sequencing analyses, we selected 10 DEGs for qRT-PCR using samples of leaves, stems, roots and bulbs originally used for RNA-Seq, all of which are known to be related to cold stress, including the genes encoding *LlAP2* (putative AP2/EREBP transcription factor, KJ489026), *LlLEA* (late embryogenesis abundant protein, KJ489024), *LlCIS* (peptidyl-prolyl cis-trans isomerase, KJ467624), *LlDREB1* (dehydration-responsive element binding, KJ467618), *LlHOT* (heat shock protein, KJ467620), *LlCOR12* (cold-regulated LTCOR12, KJ489025), *LlMYBR* (MYBR domain class transcription factor, KJ467623), *LlNAC1* (NAC domain protein, KJ467622), and *LlCDPK* (calcium-dependent protein kinase, KJ467621), *LlGPAT* (KJ467617). The Ct values of the *LlTIP1* rRNA in all samples ranged from 24.0 to 26.0. All 10 transcripts showed the same expression pattern as the *in silico* differential analysis results from high-throughput sequencing. We have provided GenBank accession numbers for our gene nucleotide sequences in Table 4 and Additional file 2: Txt S3.

**Table 4:** **Primers used in real-time quantitative PCR of** ***Lilium lancifolium*** **(RT-qPCR)**

Unigene Id	Gene name	Annotation	Forward primer sequence (5′-3′)	Reverse primer sequence (5′-3′)	Correlation between RNA-Seq and qRT-PCR(r ^2^ )	GenBank accession numbers
Contig15860	LlAP2	Putative AP2/EREBP transcription factor	CCGCCCTCTTCAATCTCATC	TATCTGGCTCGGCTCCTAC	0.98	KJ489026
Contig18777	LlLEA	LEA-like protein	AAGATGTTCCTCCTCGTGTTG	GATGTTGGTCCTCGCCTTC	0.99	KJ489024
Contig11020	LlCIS	Peptidyl-prolyl cis-trans isomerase	TTGTTCCTTCCACCGCATTA	AAAGCCTTCATCCTCAAACTTAGAC	0.95	KJ467624
Contig9406	LlDREB1	DRE-binding protein	AAATCCGCCTCCCCAAGAA	AGTTGAGCCGAGCGAAGT	0.98	KJ467618
Contig1500	LlHOT	Heat shock protein	ATGATTGGGAGGAGCACTTG	GAAGACACGGCGGACATAA	0.97	KJ467620
Contig13202	LlCOR12	Cold-regulated LTCOR12	CGGACACAACTTGACTCTTACC	CTTGCTATGCCTCGCTGAC	0.94	KJ489025
Contig1641	LlMYBR	MYBR domain class transcription factor	TTCCTCAGTCCACGCTATCC	GCCGTTGCCTAACTACTTGTC	0.98	KJ467623
Contig25399	LlNAC1	NAC domain protein	GGTTTAGAGGGAGGTTGGAGAAG	CGACGACACTGGCTCATCA	0.94	KJ467622
Contig22048	LlCDPK	Calcium-dependent protein kinase	GTCGTGCTCCAATTACCAGAAG	CAAGAGGAACAACATCACCAGAC	0.94	KJ467621
Contig5309	LlGPAT	GPAT	TGCAAAGTGGAAATCCTAATGCGAG	AGGATCTGCTTCCGTCTGATGGTTT	0.97	KJ467617
	LlTIP1	Reference gene	CGAAGCCAGAAACGGAGAAGAAT	GGGTAGGGTGGATTGGGAAGA		

**Figure 6:**
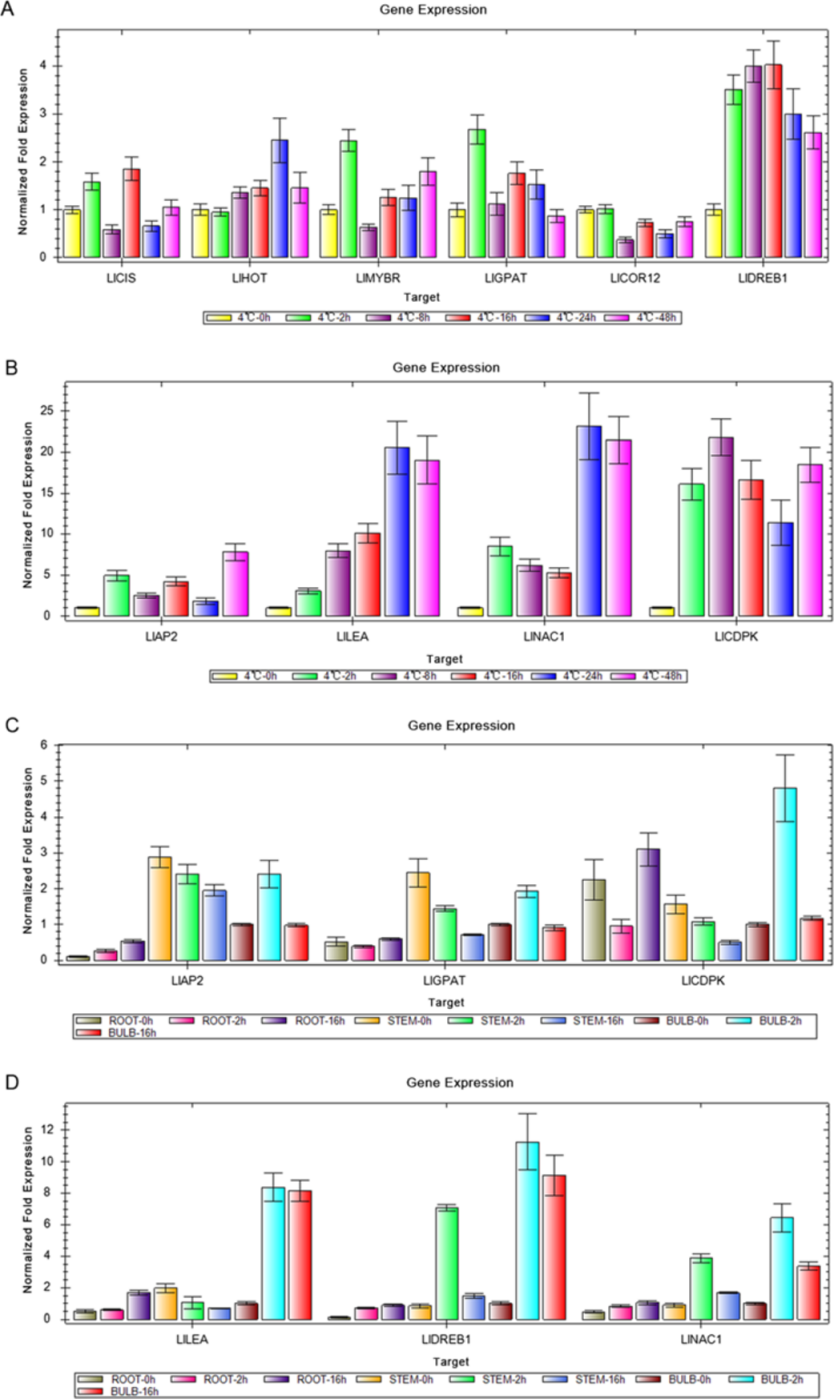
**The expression profiles of 10 transcripts in** ***Lilium lancifolium*** **by the quantitative reverse transcription polymerase chain reaction (qRT-PCR).** The Figures **A** and **B** indicated the expression of ten genes from their leaves; the Figures **C** and **D** implied the expression of six genes from their stems, roots and stems. The y-axes show normalized fold expression levels determined by qRT-PCR.

These genes were selected for their key roles in regulating stress signal transcription, cold responses, and cold acclimation. The results presented in Figure 6A and 6B showed that the expression levels of six genes were higher during CT2h to CT16h than in the other stages, including *LlCIS*, *LlMYBR*, *LlGPAT*, *LlCOR12*, *LlDREB1* and *LlCDPK*, indicating their signal transduction and transcriptions reaction after receiving the cold signal. Four genes, encoding *LlAP2*, *LlLEA*, *LlNAC1* and *LlHOT,* were more highly expressed between 24 h and 48 h than in the initial stages, and showed higher expression values than the former six genes (Figure 6A B), demonstrating that these genes may react slowly after the transcription factors and are related to cold acclimation. Also, the expression levels of the *LlLEA*, *LlNAC1*, *LlCDPK* and *LlDREB1* genes were highest in bulb at 2 h and 16 h than in the other tissues, including roots and stems and reached a higher value in the stem CT2h sample. The *LlCDPK* genes were remarkably highly expressed in roots at 16 h (Figure 6C, D). We predicted that the bulb has played an important role in for the *L. lancifolium* to resistance and adaptation to cold stress. These results indicated that there was a close correlation between the expression changes (fold difference) measured by RNA-Seq and those by qRT-PCR (Table 4).

### Soluble protein, starch, soluble sugar and Malondialdehyde concentration

**Figure 7:**
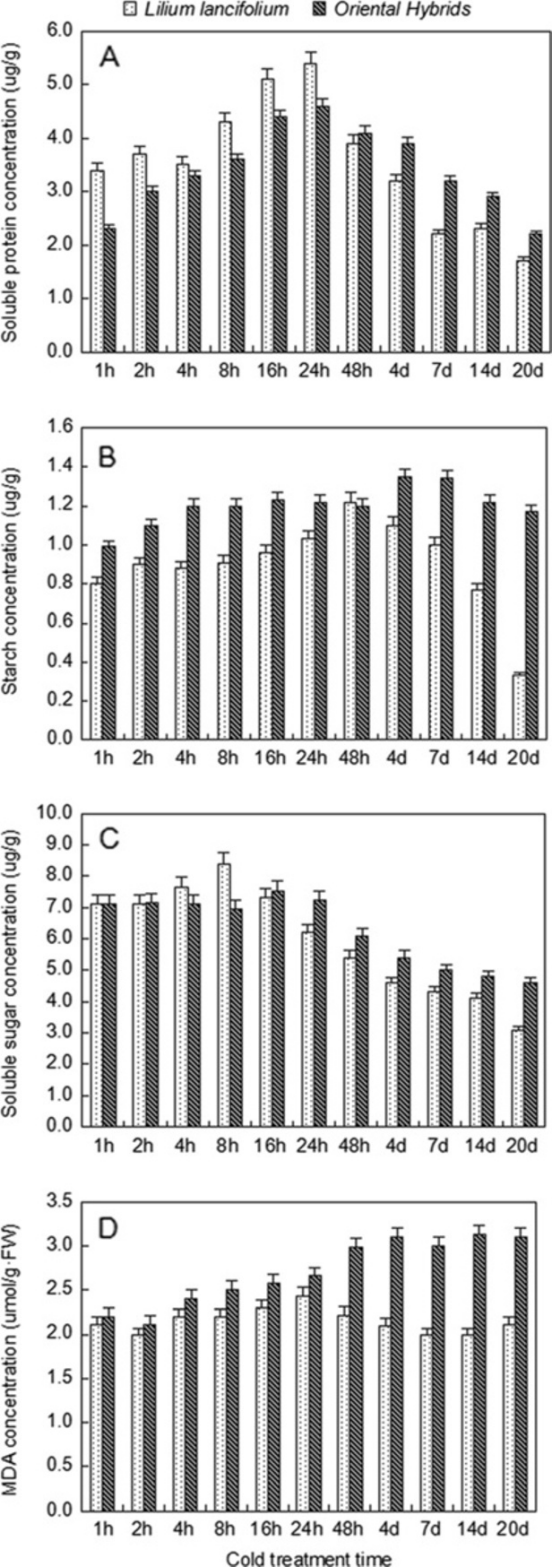
**Soluble sugar, starch, soluble protein and MDA concentration per unit 4°C cold treatment (CT) of** ***Lilium lancifolium*** **and Oriental hybrids** ***’*** **leaves. A**. Two cultivars’ soluble protein content in different stages under 4°C cold treatments **B**. Two cultivars’ starch content in different stages under 4°C cold treatments **C**. Two cultivars’ soluble sugar content in different stages under 4°C cold treatments **D**. Two cultivars’ MDA content in different stages under 4°C cold treatments Black squares: *Lilium lancifolium*; grey squares: Oriental hybrids’fructose; Bars refer to standard errors.

To elucidate the mechanism underlying the cold response, it is important to determine how plants alter gene expression in response to this biological process. The levels of two measured soluble proteins increased sharply and rapidly following cold exposure of *L. lancifolium* and Oriental hybrid leaves, but the soluble protein levels of *L. lancifolium* declined more sharply and faster than those of the Oriental hybrids from 2 d to 14 d of cold exposure, and the totals of the two varieties showed significant differences that reached a maximum at 24 h (Figure 7A, Additional file 1: Table S2). On an area basis, starch tests showed that *L. lancifolium* leaves had increasing starch content between 1 h to 48 h of cold treatment, which rapidly fell during subsequent cold stress; although unlike *L. lancifolium,* the cold development slowly and incrementally increased the starch content of the Oriental hybrids leaves (Figure 7B, Additional file 1: Table S2)*.* Soluble sugar levels also decreased, with linear regression significant on a CT basis, and exhibited a greater decrease in *L. lancifolium* than in *the* Oriental hybrids when measured at 4°C (Figure 7C, Additional file 1: Table S2); however, the data suggested a peak at 16–24 h. Total nonstructural carbohydrates (TNCs) responded non-linearly, because of the influence of starch and glucose. Both in *L. lancifolium* and Oriental hybrid leaves*,* cold development increased MDA levels relative to the cold response from 1 h to 16 h, whereas, MAD content in *L. lancifolium* leaves declined significantly with increasing cold exposure duration at 4°C in contrast to the increase in the Oriental hybrids during the same stage (Figure 7D, Additional file 1: Table S2). The *L. lancifolium* leaves responded more strongly than Oriental hybrid leaves during the cold treatment.

### Membrane systems and cellular osmoprotectant

**Figure 8:**
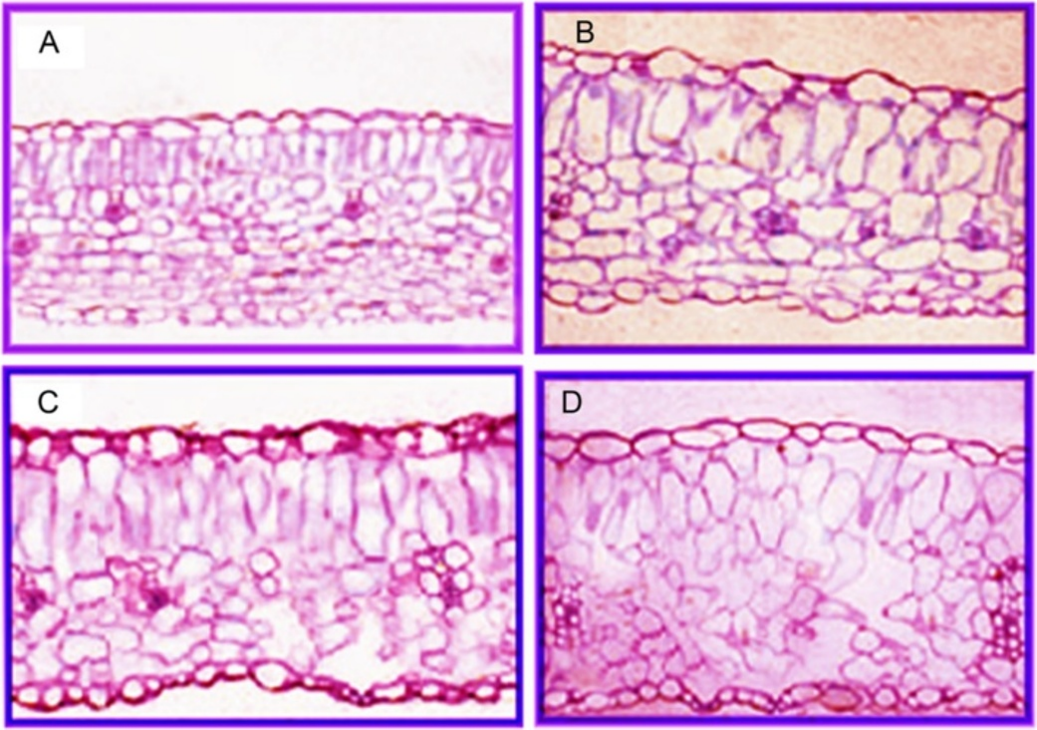
***Lilium lancifolium*** **leaf structures in different stages under 4°C cold treatments. A**. Controlled 0 h(grow up in room-temperature) leaf structure; **B**. cold treatment 16 h leaf structure; **C**. cold treatment 48 h leaf structure; **D**. cold treatment 7 days leaf structure.

Membrane systems, which are known to be the primary site of freezing injury in plants, suffer multiple forms of damage caused by freeze-induced cellular dehydration [22]. During cold acclimation, plants experience improved cold tolerance with increased cellular metabolic activity, and increased concentrations of unsaturated fatty acids and phospholipids [23]. Correspondingly, we identified a total of 1153 genes (4.46%) involved in 'Lipid transport and metabolism’ and 3333 genes (7.01%) involved in 'Intracellular trafficking, secretion, and vesicular transport’ according to the COG classification. Furthermore, according to the metabolic pathway enrichment analysis, the 'biosynthesis of unsaturated fatty acids’ (ko01040), 'fatty acid elongation’ (ko00062) and 'regulation of actin cytoskeleton’ (ko04810) pathways were all involved in lipid metabolism. Increases in the biosynthesis of unsaturated fatty acids improve cold defense and prevent damage caused by low temperatures [24]. Thirty genes in this process showed significant regulation of their transcripts after cold stress. For example, two chloroplast omega-3 fatty acid desaturase genes (Contig10730_All, Contig17187_All) were up-regulated by 0.38 to 1.15-fold. The FAD3 gene in *L. lancifolium* encodes a chloroplast membrane-associated omega-3 fatty acid desaturase, which contributes to freezing tolerance by altering the lipid composition [25]. In our present study, the analysis of the structural characteristics of *L. lancifolium* leaf cells showed that leaf thickness doubles compared with room-temperature controls during cold treatment. This thickening is a result of palisade cell length elongation and an enlargement of the intercellular spaces caused by a more loosely packed spongy parenchyma matrix [26]. The intercellular spacing and spongy parenchyma packing were homoplastically altered following the cold treatment, they were close together at 16 h, but the palisade cell length and spacing increased prior to 48 h, and was most altered after 7d of treatments (Figure 8).

**Figure 9:**
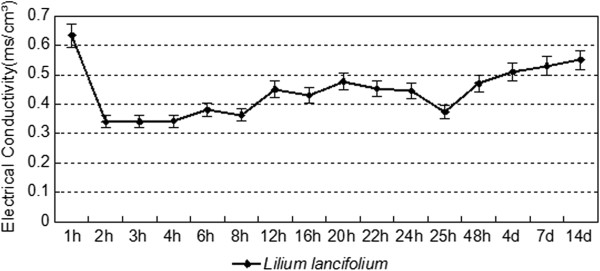
**Electrical conductivity expression of** ***Lilium lancifolium*** **leaves in different stages under 4°C cold treatments.**

Proline, one of the most effective organic osmolytes, participates in responses to various environmental stresses [27]. Moreover, according to the metabolic pathway enrichment analysis, 'proline metabolism’ (ko00330) involved 69 genes that regulate and relieve the osmotic stress caused by cold-induced dehydration. Two genes (Contig7616, Contig7617) for delta1-pyrroline-5-carboxylate synthetase (P5CS) were significantly up-regulated by 0.88 and 1.29-fold after cold stress, respectively. As a key enzyme in proline synthesis, DREB1 participates in the cold-stress response and shows high expression, which promotes the synthesis of proline for cold tolerance [28]. The accumulation of sucrose and other simple sugars also contributes to the stabilization of membranes, as these molecules protect membranes against freeze-induced damage in vitro. Five *LEA* (late embryogenesis abundant)-related genes (Contig24352_All, Contig19954 _All, Contig17189_ALL, Contig26956, Contig18777) were also differentially expressed with fold changes in their expression ranging from 0.23 to 1.30-fold. The LEA protein functions as an antioxidant, as well as a membrane and protein stabilizer, during cold stress [29]. Our studies have analogously suggested that the electrical conductivity of *L. lancifolium* leaves decreases at 2 h after but increases at 12 h, and then keeps a positive slope until 24 h cold treatment It is reasonable to infer that hydrophilic and LEA polypeptides stabilize membranes against freezing-induced injury in *L. lancifolium*[30]. Proline levels further enhanced the electrical conductivity of *L. lancifolium* leaves in different stages under 4°C cold treatment (Figure 9). The electrical conductivity dropped at 2 h, but kept gradually increasing with prolonged cold treatment. It can be inferred that the accumulation and effects of Proline may contribute to a remarkable control of cellular electrical conductivity, which reflects the destruction of cell wall and cytoplasm motivation, furthermore regulating outstanding cold tolerance for *L. lancifolium* (Figure 9).

## Discussion

### Illumina paired-end sequencing, assembly, and functional annotation

Transcriptome analysis is important for elucidating the molecular constituents of cells and tissues and interpreting the functional elements of the genome [31]. In our study of the transcriptome of *L. lancifolium* (3n = 27), we sampled the pooled transcriptomes of leaves using Illumina paired-end sequencing technology to generate a large-scale EST database. Approximately 10.7 GB of data was generated and assembled into 37,843 unigenes. This large number of reads with paired-end information produced much longer unigenes (mean, 973 bp) than those in other lily studies. This increased coverage depth of the transcriptome facilitated *de novo* assembly, enhanced sequencing accuracy, and avoided possible contamination. Of the 37,843 *L. lancifolium* unigenes, 18,736 (49.30%) had homologs in the Swiss-Prot database. More importantly, we were able to assign a number of these unigenes to a wide range of GO categories and COG classifications (Figures 1 and 2), indicating that diverse transcripts are involved in the cold response and which are represented in the sequence data of this species, and also reflecting the complexity of differential low temperature signal transcription in *L. lancifolium*.

Furthermore, during cold responses and tolerance, plants receive low temperature signals and initiate a defense mechanism, including physical structure adaptations (changes in lipid composition and extracellular metabolic activity), increases in intercellular osmoprotectants (such as soluble sugars, proline and betaine), and up-regulated synthesis of anti-oxidants (superoxide dismutase, pathogen defense, catalase and ascorbic acid reductase), enabling restoration of the balance of biosynthesis and carbohydrate metabolism and enhancing survival in cold environments [32]. Interestingly, part of the expression patterns of a large number of genes during cold stress stimuli and transcription factors have been detected using transcriptome sequencing and microarray technologies. Most representative unigenes were annotated to specific pathways, such as metabolic pathways, biosynthesis of secondary metabolites, microbial metabolism in diverse environments, the mRNA surveillance pathway, ribosomes, pyrimidine metabolism, biosynthesis of amino acids, the cell cycle, carbon metabolism and plant hormone signal transduction using the KEGG databases (Table 3), leading us to conclude that most of the genes we identified are involved in the cold response and signaling regulation.

### Carbohydrate metabolism

A principal factor in carbohydrate metabolism under stress conditions is regulation of the balance between biosynthesis and breakdown of proteins. Proteolysis plays a dynamic and vital role in the regulation of different metabolic processes, and in the cell’s response to environmental stimuli. It controls metabolic fluxes by regulating the levels of key rate-limiting enzymes, while also irreversibly irreversibly polypeptides into soluble sugars that may interfere with these pathways. Much of this directed protein turnover is performed by proteases that require ATP and the Clp protease is one of the best characterized to date [33]. Through our KEGG analysis, we discovered 186 regulated genes in the 'Starch and sucrose metabolism’ pathway and 208 regulated genes in the 'Protein processing in endoplasmic reticulum’ pathway (Table 3). Moreover, the protein of *L. lancifolium* increased with the rapid cold stimulus and then degraded into sugars with a faster reduction trend than the Oriental hybrids during the cold treatment. This demonstrated that *L. lancifolium* could adapt to 4°C cold treatment better than the Oriental hybrids, by accumulating protein and converting it into sugar; increasing the cell liquid concentration and reducing the freezing point, so as to prevent the frost damage (Figure 7A, Additional file 1: Table S2). This supports the fact that *L. lancifolium* can acclimate to a cold environment and elicit a series of physiological and biochemical responses to low temperature, such as the transformation and combination of soluble sugars (Figure 7C, Additional file 1: Table S2). The reason for the difference is that Oriental hybrids grow in slightly lower latitudes than *L. lancifolium* and their optimum growth temperature is 25°C. Interestingly, in the *L. lancifolium* transcriptome*,* we found related proteolytic enzymes, including an ATP-dependent Clp protease proteolytic subunit, with a fold change of 0.94 (down-regulation) from 0 h to 2 h but a fold change of 1.28 (up-regulation) during 2 h to 16 h (Contig24056_All), a Clp-like energy-dependent protease, which showed a 1.56 fold change (Contig1979_All), and also Sucrose phosphate synthase and Glucose-6-phosphate 1-dehydrogenase, which were up-regulated with 1.67 and 1.12 fold changes, respectively (Contig7878_All, Contig3504_All). Overall, the data suggested that transfer of *L. lancifolium* leaf soluble protein is crucial for determining soluble synthesis, and induces better cold resistance than Oriental hybrid leaves, which cannot respond and acclimate to low temperature (Figure 7C, Additional file 1: Table S2).

The enzymes Alpha-amylase, β-amylase and Isoamylase degrade starch to soluble sugar, the further conversion of which leads to increased maltose, glucose, fructose and sucrose levels [34]. The effects of CT and cold duration on the expression of alpha-amylase, β-amylase, and isoamylase in *L. lancifolium* were shown by Illumina sequencing, with 2.25-fold up-regulation from 0 h to 2 h and 0.93-fold down-regulation from 2 h to 16 h, 0.73-fold up-regulation from 0 h to 2 h; and 1.78-fold up-regulation from 0 h to 2 h and 0.70-fold down-regulation, from 2 h to 16 h, respectively (Contig5436_all, First_Contig433_all, Contig13233_all). Thus, it is unsurprising that a subset of starch hydrolysis enzyme genes display a highly specific, largely up-regulation response between 0 h to 2 h of 4°C cold stress, because hydrolysis enzymes affect the degradation of carbohydrate during the early period of cold stress [35]. On an area basis, starch tests showed that the starch content of *L. lancifolium* leaves had increased between the 1 h to 48 h of cold treatment, and fell during subsequent cold stress as it was degraded into soluble sugar. The limited starch degradation of Oriental hybrid leaves indicated its poor ability to resist and adapt to cold exposure (Figure 7B, Additional file 1: Table S2)*.* In *L. lancifolium*, we detected that the expression of 47 soluble sugar synthase DEGs out of 1326 different enzyme DEGs was significantly and markedly induced by 2 h and 16 h cold stress, including five up-regulated and 19 down-regulated glucose synthase genes, two up-regulated and four down-regulated fructose synthase genes and three up-regulated and 14 down-regulated sucrose synthase genes. Thus, exposure to 4°C cold treatment clearly favored starch degradation, which would result in increased accumulation of soluble sugars. In addition to the effects of chilling on β-amylase transcript levels, we found that CT increased the expression levels of a specific Sucrose-phosphate synthase7 (SPS7) transcript (First_Contig33) by 0.73-fold. Because SPS1 catalyzes sucrose synthesis, it is possible that increased accumulation of sucrose in *L. lancifolium* may contribute to cold tolerance (Figure 7B, C, Additional file 1: Table S2).

MDA (malondialdehyde) reflects the ability of plants to resist and acclimate to abiotic stress [36]. In *L. lancifolium* under 4°C cold treatment, MDA showed no obvious change during the early cold period but declined during the 48 h to 20 d period, which demonstrated the better cold resistance characteristics of in *L. lancifolium* compared with the Oriental hybrid (Figure 7D, Additional file 1: Table S2). To some extent, it is possible that MDA is related with proline, and the up-regulation of the delta1-pyrroline-5-carboxylate synthetase (P5CS, Contig7616_all, 1.29 fold) would enhance proline concentration to strengthen the plant’s osmotic adjustment ability, resulting in the mitigation of cellulose peroxidation.

### Signaling pathways

**Figure 10:**
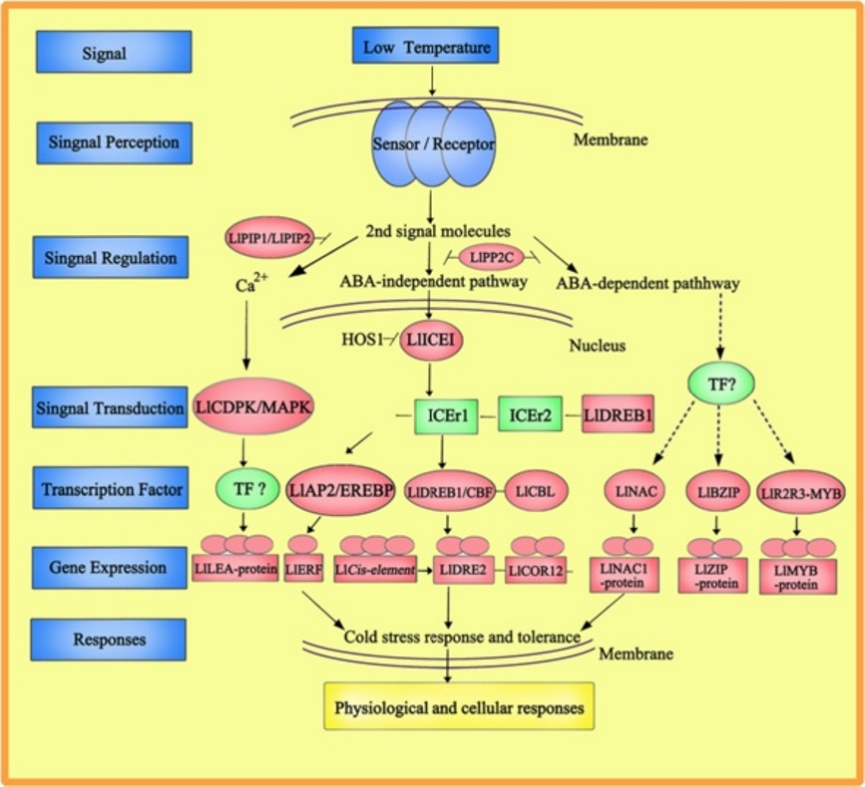
**Models describing the signaling pathways involved in the acquisition of cold tolerance.** The red box showed the indentified genes in *Lilium lancifolium* transcriptome; and the blue box demonstrated the unknown transcription factors and pathways in *Lilium lancifolium.*

In endocrine signaling, hormones act on distant target cells in paracrine signaling, a molecule released from a cell acts on nearby targets and in autocrine signaling, a cell produces a signaling molecule to which it also responds [36]. The binding of most signaling molecules to their receptors initiates a series of intracellular reactions that regulate virtually all aspects of cell behavior, including metabolism, movement, proliferation, survival, and differentiation. Understanding the molecular components of these pathways and how they are regulated has thus become a major area of research in contemporary cell biology [37]. Two major pathways of intracellular signaling are based on the use of second messengers derived from the membrane phospholipid phosphatidylinositol 4,5-bisphosphate (PIP). PIP_1_ and PIP_2_ are minor components of the plasma membrane, localized to the inner layer of the phospholipid bilayer. A variety of hormones and growth factors stimulate the hydrolysis of PIP by a phospholipase C-a reaction that produces two distinct second messengers: diacylglycerol and inositol 1,4,5-trisphosphate(IP_3_), which can stimulate distinct downstream signaling pathways (protein kinase C and Ca^2+^ mobilization, respectively), so that PIP_1_ and PIP_2_ hydrolysis triggers a two-armed cascade of intracellular signaling. In the *L. lancifolium* trancriptome, six genes in the PIP process showed significant transcript regulation after cold stress (Figure 10). For example, two Plasma membrane intrinsic protein genes (Contig1854_All, Contig1406_All) were up-regulated by 0.58 and 1.36-fold. Thus, the *LlPIP*_*1*_ and *LlPIP*_*2*_ genes in *L. lancifolium* encode plasma membrane intrinsic proteins, which contribute to triggering the protein kinase C and stimulating Ca^2+^ signaling pathways in response to low temperature [38].

Here, in *L. lancifolium*, protein kinase C including the receptor protein kinase *LlCLAVATA1*, a protein kinase Ck2 regulatory subunit and 2Serine/threonine-protein kinase cdk9 (Contig18123_All, Contig8528, Contig14527), showed up-regulated expression from 0.33- to 1.01-fold during the cold treatment, bridging the process of signal transduction. Another two contrasting themes are apparent in the expression of the signaling pathways initiated by Ca^2+^ and cAMP. These two second messengers may operate towards a similar goal. The activation of phosphorylase kinase through either PKA or Ca^2+^ is an example of such convergence. Ca^2+^ and cAMP-mediated pathways may also be coordinated through Ca-calmodulin dependent isoforms of adenylyl cyclase [36]. As an important second messenger, Ca^2+^ plays a vital role in the plant cold-stress response. The concentration of Ca^2+^ increases rapidly during cold stress, followed by a number of signals mediated by combinations of protein phosphorylation cascades [39]. As a large subfamily of plant kinases, Calcium dependent protein kinases (CDPKs) are implicated as important sensors of Ca^2+^ flux in plants in response to a variety of biotic and abiotic stress stimuli [40]. We identified two genes (Contig22048_All, Contig2751_All) related to CDPK, with fold changes ranging from 0.89- to 3.28-fold in their expression after cold stress (Figure 9); they have been demonstrated to activate a stress and ABA-inducible promoter. These results demonstrate the connection of particular CDPKs to specific signaling pathways *in vivo* and the usefulness of engineering CDPKs to enhance abiotic stress tolerance in *L. lancifolium* (Figure 10).

In many plant cells, Abscisic acid (ABA) also plays a crucial role in the cold tolerance of plants. The type 2C protein phosphatases (PP2C), which negatively regulate ABA responses, play a key role in ABA signal transduction [8, 39]. In this study, two DEGs (Contig24025_All, Contig19208_All) related to PP2C were identified that showed significant down-regulation, with fold changes ranging from 0.95 to 0.35, in their expression after cold stress. The cold response has been reported to involve both ABA-dependent and independent pathways [41]. In the ABA-independent pathway, the transcription factor of DREB1 (DRE-binding protein) has also implicated in dehydration stress signaling in Arabidopsis [42]. In our research, one gene (Contig9406_All) related to *LlDREB1* was identified, with a fold change of 0.31. We presume it would trigger and induce the expression of *LlCOR12* and *LlDRE2* (Contig13202_All, Contig12185_All), in combination with the up-regulation of the *LlCIS*-element (Contig8665) by a change of 1.51-fold. One of the big gene families that have been investigated in our study not only includes the *LlAP2/EREBP* (Contig10652_All) transcription factor, but also cold genes such as *LlERF2*, *LlERF3*, *LlERF5*, *LlERF10* and *LlmTERF* (Contig18905_All, Contig772_All, Contig15936_All, Contig26562_All, Contig28555_All). On the other hand, the ABA-dependent pathway has genes related to transcription factors; here, *LlNAC* and *LlBZIP* (Contig20596_All, Contig12014_All) in *L. lancifolium* were differentially expressed with fold changes of 0.46 and 1.56, respectively, subsequently motivating the expression of the *LlNAC1* and *LlZIP* proteins after cold exposure for 16 h. Moreover, we also obtained a large amount of information on the MYB family from *L. lancifolium* Illumina sequencing, including the chief transcription factors *Ll1R-MYB1*, *LlR2R3-MYB and LlMYBR* (Contig22140_All, Contig18508_All, Contig1641_All), which showed fold changes of 1.01, 6.30, and 1.85, respectively, and stimulated the identified cold gene *LlMYB-DNAbinding protein* up-regulation with a 2.92-fold change (Contig11131_All). Therefore, it is likely that the *LlDREB1*, *LlCBL*, *LlAP2/EREBP*, *LlNAC*, *LlBZIP* and *LlR2R3-MYB* genes of *L. lancifolium* show transcription patterns under cold stress similar to those under other abiotic stresses, which are believed to activate the transcription of specific target genes in ABA signaling in guard cells (Figure 10).

Whereas the cells of prokaryotes and unicellular eukaryotes are largely autonomous, the behavior of each individual cell in multicellular plants must be carefully regulated to meet the needs of the organism as a whole. This is accomplished by a variety of signaling molecules that are secreted or expressed on the surface of one cell and bind to receptors expressed by other cells, thereby integrating and coordinating the functions of the many individual cells that make up the complex organisms [37]. In this case, cells receive and respond to signals from a cold environment, and then cold signals are received by sensors on the membrane and the second signal molecules carry them from the cytoplasm to the nucleus by the catalytic protein kinase regulatory subunit. Within the nucleus, protein kinase C and PP2C induce the Ca^2+^ pathway and ABA signal transduction, respectively, and various transcription factors such as *LlDREB1*, *LlAP2/EREBP*, *LlNAC*, *LlBZIP* and *LlR2R3-MYB* recruit coactivators for the transcription of inducible genes including *LlLEA LlERF*, *LlDRE2*, *LlNAC1*, *LlZIP* and *LlMYB-DNAbinding* protein, which are regulators of cellular cold tolerance and metabolic activity in short-term cold stress, and *LlFAD3*, *Llβ-amylase* genes, *LlP5CS* and *LlCLS,* which enhance adaptation processes that involve changes in the expression of transcripts related to cellular osmoprotectants, and carbohydrate metabolism during the long-term cold stress. Such regulation of gene expression plays important roles in controlling the physiological cold response, cellular morphology, proliferation, survival, and differentiation of a wide variety of plant cells, as well as being implicated in learning and memory (Figure 10).

To further study the mechanism of the cold response and acclimation of *L. lancifolium,* we will select specific genes and verify their functions and expression by fluorescence in situ hybridization and genetic modification technology.

## Conclusions

To the best of our knowledge in *L. Lancifolium,*, interest is further heightened by the fact that stress-regulated genes are stimulated and reacted during the short-term cold response, along with the physiological and biochemical changes during the long-term cold acclimation. The genome-wide transcriptome and physiological analysis presented in this study has expanded our knowledge of this process by identifying differentially expressed genes involved in cold regulation of carbohydrate metabolism, leaf structure and three model signaling pathways, the Ca^2+^ and ABA-dependent/independent pathways. Importantly, the high-resolution expression patterns presented here further our understanding of the molecular mechanisms involved in cold resistance and signal regulation for bulb flower breeding.

## Methods

### Ethics

This research did not involve any human subjects, human material, or human data. The field study did not involve any endangered or protected species.

### Plant material and total RNA isolation

The materials used in these experiments were derived from *L. lancifolium*’s and Oriental hybrids cultivated in the nursery of Beijing Forestry University (BJFU) (116.3°E, 40.0°N) under the following growth conditions: 70% relative humidity, 25°C:18°C, day:night temperatures, with watering every 3 days. The bulbs were collected and stored at 4°C for one month. Then, the bulbs were cultivated under aseptic conditions to induce leaf formation. After 4 weeks of asepsis condition, we divided the plantlets into two groups; the control sample (CK0h) and the cold-treated sample (CT). Fresh leaves, stems, bulbs, and roots were subjected to a 4°C cold treatment for 2 h, 8 h, 16 h, 24 h, 48 h, 4 d, 7 d, 14 d, or 20 d. At each time point, samples were collected and stored at -80°C until RNA extraction. Total RNA was extracted from the tissues using an RNAisomate RNA Easyspin Isolation System (Aidlab Biotech, Beijing, China) according to the manufacturer’s instructions. The quality of RNA was verified using a 2100 Bioanalyzer (Agilent Technologies, Santa Clara, CA, USA). The RNA concentration was at least 160 μg/mm^3^ in all samples. To prepare cDNA, we used a pooled RNA mixture containing 60 μg RNA from each sample.

### cDNA library preparation and transcriptome sequencing

Illumina sequencing was conducted using the Solexa mRNA-Seq platform at the Shanghai manufacturer’s instructions (Illumina, San Diego, CA, USA). Briefly, we used magnetic beads with oligo(dT) to isolate poly(A) mRNA after isolating total RNA from *L. lancifolium* leaves in the control (0 h) and after 2 h and 16 h of cold treatment. Second-strand cDNA was synthesized using appropriate buffers, dNTPs, RNase H, and DNA polymerase I. ShoBiotechnology Corporation (SBC), Shanghai, China (http://www.ebioservice.com) according to the rt fragments were depurated with a QiaQuick PCR extraction kit (Qiagen, Hilden, Germany) and resolved with an elution buffer for end repair and by addition of poly(A). For PCR amplification, we selected suitable fragments as templates based on the results of agarose gel electrophoresis. The library was sequenced using an Illumina HiSeq™ 2000. Because raw reads produced from sequencing machines contain low-quality reads that negatively affect subsequent bioinformatics analyses, we discarded reads with adapters, those with more than 5% unknown nucleotides, and those of low quality (≤ 20% of the bases with a quality score (Q) ≤ 10) using an in-house Perl script. The average proportion of clean reads in each sample was 89.6%–91.7%. The clean reads were used for further analyses.

### Analysis of Illumina transcriptome sequencing results

*De novo* assembly was carried out using scaffolding contig methods with CLC Genomics Workbench (version: 5.5) with the default parameters, and a minimum contig length of ≥400. The assembled *de novo* sequences were designated as primary unigenes. After assessing the different K-mer sizes, we found that 29-mer yielded the best assemblies and so this size class was selected to construct the de Bruijn graph. Primary unigenes from UniGene of three samples’ were assembled using CAP3 ES, yielding final unigenes. Assembled final unigenes were used for BLASTx searches (E-value <1e-5) against the UniProt database (date: 2013.04) and the Swiss-Prot protein database (date: 2013.05) (http://www.expasy.ch/sprot), which has the largest and most particular protein annotation database (approximately including 24,889,084). To functionally annotate sequences, we used Blast2GO program (Conesaet et al., 2005) to assign gene ontology (GO) terms (http://www.geneontology.org). Also, to predict and classify possible functions, 13705 unigene sequences were aligned to 25 Clusters of Orthologous Groups (COGs) in the COG database (http://www.ncbi.nlm.nih.gov/COG). Kyoto Encyclopedia of Genes and Genomes Pathway (KEGG; http://www.genome.jp/kegg) annotations were carried out according to the KEGG database using BLASTx (E-value threshold 10^-5^).

### Bioinformatics for functional annotation of differential gene expression

A rigorous algorithm to identify differentially expressed genes was developed based on the method of Audic et al. (1997). The false discovery rate (FDR) was used to determine the threshold of the P-value in multiple tests and analyses. We used an FDR of < 0.001 and the absolute value of log_2_ (ratio) ≥ 2 as thresholds to define significantly different gene expression [43]. For further analyses, we used an additional criterion, which involved using only differentially expressed genes (DEGs) with a minimum of a four-fold change in expression.

### Transcription factors analysis

Transcription factors were predicted according to protein sequences obtained from CDS predictions. We used HMM search to search for plant transcription factor domains (http://plntfdb.bio.uni-potsdam.de/v3.0/) and classified unigenes according to gene family information.

### Real-time quantitative PCR verification

Total RNA was isolated from the leaf, stem, bulbs, and roots of lily plants subjected to 4°C cold treatments, as described above. First-strand cDNA synthesis was performed using Superscript II reverse transcriptase (Invitrogen, Carlsbad, CA, USA) according to the manufacturer’s instructions, using 1 μg total RNA and oligo(dT) primers. qRT-PCR was performed using a Rotor-Gene 3000 real-time PCR detection system (Qiagen) using SYBR® qPCR Mix (Toyobo, Tokyo, Japan) according to the manufacturer’s protocol. The primers used in this study were designed with Beacon Designer (Premier, Palo Alto, CA, USA) and are listed in Table 1. Real-time PCRs was carried out using prepared cDNA (80 μg) with each set of primers and probe and iQ™ SYBR® Green Supermix (Cat. No.170-8882, Bio-Rad, Hercules, CA, USA). The PCR cycling conditions were as follows:95°C (30 s), 60°C (30 s), and 72°C (15 s). All reactions were performed in biological triplicates. Relative mRNA levels were calculated using the 2^-△△Ct^ method [44] against the internal reference gene *TIP1*, with expression in CT 0 h used as the internal control. The sequences of primers used for QRT-PCR are listed in Table 4.

### Heat-map generation

A heat-map of legume-specific genes and the genes with the highest transcript levels was generated using the heat-map function in the gplots CRAN library. After excluding legume-specific genes that did not have a RPKM normalized log_2_-transformed transcription count greater than zero in at least one tissue, 315 genes remained. The LSGs were taken from the Glyma1.01 gene set. The genes with the highest transcript levels were determined based on the sum of raw counts in all tissues. Boxes were added to reveal clusters of genes with similarly expression in specific tissues. (Additional file 1: Table S1) showed additional details indicating the gene represented by each cell in the heat-map.

### Leaf structural characteristics

To investigate the internal anatomy of leaves, sections were cut through the leaf midrib. The proximal halves of individual leaves were fixed in 0.3 mg/cm^3^ paraformaldehyde, 5% ethanoic acid, and 50% ethanol, and then dehydrated in a graded ethanol (50%–95%) series. Sections (1 μm thick) were cut with a micrometre (Ultracut UCT, Leica Microsystems,Welzlar, Germany), stained with toluidine, and imaged with a microscope and imaging system (Optiphot 2 with DS-L1, Nikon, Tokyo, Japan). The cut surface was mid-way between the midrib and margin, near the widest point of the leaf.

### Carbohydrate and electrical conductivity analysis

Total soluble proteins, soluble sugars, starch, and malondialdehyde (MDA) content were determined using leaf tissue from plants subjected to 1 to 20 days of cold treatment. The leaf tissue was collected and stored at -80°C. Carbohydrate content and electrical conductivity were measured as described by Gilmour [45]. Soluble sugars were analyzed using the phenol-sulfuric acid method. Soluble proteins were determined using the Coomassie brilliant blue colorimetric method. Starch was quantified using the anthrone-sulfuric acid method and MDA content was determined using the thiobarbituric acid method. Absorbance was measured using a plate reader (POLARstar OPTIMA, BMG Labtech, Offenburg, Germany). Electrical conductivity was measured by the bath method using a desktop meter (EC3175-307, JENCO, San Diego, CA, USA).

### Availability of supporting data

The data sets supporting the results of this article are available in the [NCBI GenBank] repository, [unique persistent identifier (KJ467617, KJ467618, KJ467619, KJ467620, KJ467621, KJ467622, KJ467623, KJ467624, KJ489024, KJ489025, KJ489026,) and hyperlink to datasets in http://www.ncbi.nlm.nih.gov/genbank/].

And also, the other data sets supporting the results of this article are included within the article and its additional files TXTS3.

## Electronic supplementary material

Additional file 1: **Table S1.** Differential expression genes in the heat-map.Heat-map of 65 differentially expressed genes involved in transcription factor, signal transport, stress kinase, defense/stress response, target protein compound in the cold response and acclimation of *Lilium lancifolium*. **Table S2.** The analysis of variance (ANOVA) of different physiology measurements for *L. Lancifolium*. (DOC 154 KB)

Additional file 2: TXT S3: The nucleotide sequences and NCBI GenBank accession numbers for the identified genes. (TXT 13 KB)

## Abbreviations

BLAST: Basic local alignment search tool’s Control 0 h
CT: Cold treatment
COG: Cluster of orthologous group
DEGs: Differentially expressed geneses Expressed sequence tag
GO: Gene ontology
KEGG: Kyoto encyclopedia of genes and genomes
MDA: Malondialdehyde
qRT-PCR: Real-time quantitative reverse transcription PCR
SP: Swiss-Prot protein database
SOM: Self-organizing map
TF: Transcription factor.
