# Supplementary material for: Transcriptome profiling of the cold response and signaling pathways in Lilium lancifolium
Source: BMC Genomics. 2014 Mar 17;15(1):203. doi: 10.1186/1471-2164-15-203 (PMC4003810; doi:10.1186/1471-2164-15-203)
Supplement: Supplementary file 1 — Additional file 1: Table S1. Differential expression genes in the heat-map.Heat-map of 65 differentially expressed genes involved in transcription factor, signal transport, stress kinase, defense/stress response, target protein compound in the cold response and acclimation of Lilium lancifolium. Table S2. The analysis of variance (ANOVA) of different physiology measurements for L. Lancifolium. (DOC 154 KB) [file 12864_2013_7041_MOESM1_ESM.doc]

**Table S1. Heat-map of 65 differentially expressed genes**

| **Category Annotation** | **Unigene_id** | **Unigene**  **length** | **Description** | **Control-0h_RPKM** | **Control-2h_RPKM** | **Treatment-16h_RPKM** | **P-value** |
| --- | --- | --- | --- | --- | --- | --- | --- |
| Transcription factor | Contig15860 | 1812 | Putative AP2/EREBP transcription factor superfamily protein | 4.871499997 | 12.33208058 | 17.16723396 | 0.006046619 |
| Transcription factor | Contig18905 | 556 | ERF2 transcription factor | 2.572529724 | 9.777928477 | 6.106988207 | 0.221033178 |
| Transcription factor | Contig10652 | 603 | Putative AP2/EREBP transcription factor superfamily protein | 1.01657891 | 1.126975173 | 2.240285337 | 0.683692133 |
| Transcription factor | Contig15936 | 983 | ERF transcription factor 5 | 14.67534556 | 18.66559795 | 11.69973326 | 0.70171262 |
| Transcription factor | Contig20596 | 504 | Putative NAC domain transcription factor superfamily protein | 16.21685405 | 15.38699926 | 35.56885459 | 0.008493575 |
| Transcription factor | Contig12014 | 998 | BZIP transcription factor ATB2 | 29.48282563 | 29.80060861 | 18.87722206 | 0.153971516 |
| Transcription factor | Contig20958 | 1226 | MYBR domain class transcription factor | 16.76658688 | 18.12871664 | 12.38858728 | 0.585549739 |
| Signal transport | Contig17439 | 780 | ABC_transporter | 19.17580618 | 13.52982139 | 107.6126418 | 1.82E-16 |
| Signal transport | Contig6966 | 1113 | Multidrug resistance protein ABC transporter family | 42.99608171 | 41.91393457 | 125.4744208 | 3.96E-11 |
| Signal transport | Contig8316 | 887 | Abc transporter, putative | 51.32497184 | 51.4215027 | 36.51059616 | 0.167838343 |
| Signal transport | Contig7293 | 3293 | MDR-like ABC transporter | 25.91229698 | 28.83066253 | 34.51487575 | 0.249145314 |
| Signal transport | Contig24166 | 1024 | White-brown-complex ABC transporter family | 2.035341877 | 0.897864124 | 5.918709924 | 0.337906514 |
| Signal transport | Contig3170 | 5009 | ABC transporter family, cholesterol/phospholipid flippase | 15.81954274 | 15.4423246 | 19.36687043 | 0.50722879 |
| Signal transport | Contig1255 | 1515 | ABC transporter B family member | 21.95729925 | 16.64943368 | 24.19580102 | 0.662657178 |
| Signal transport | Contig10729 | 3690 | Calcium-transporting ATPase 4, endoplasmic reticulum-type, putative, expressed | 13.88795559 | 14.99313541 | 35.02642558 | 0.001608345 |
| Signal transport | Contig15736 | 657 | ZIP transporter OS=Populus trichocarpa | 37.94299549 | 33.22079433 | 33.06515177 | 0.725435214 |
| Signal transport | Contig17752 | 1092 | Zinc transporter 4 OS=Gossypium hirsutum | 5.388985344 | 8.309711733 | 11.86928721 | 0.162321627 |
| Signal transport | Contig2006 | 1108 | Sugar transporter protein | 72.91788405 | 72.40863349 | 57.69860458 | 0.256649038 |
| Signal transport | Contig25518 | 623 | Sugar transporter protein | 2.886235596 | 3.015730655 | 3.926500711 | 0.723273716 |
| Stress kinase | Contig5690 | 1768 | Calcium-dependent protein kinase 1 | 22.39797034 | 27.33548474 | 47.0424989 | 0.002741998 |
| Stress kinase | Contig2751 | 1845 | Calcium-dependent calmodulin-independent protein kinase isoform 2 | 28.17460847 | 28.79461989 | 32.21639596 | 0.611915225 |
| Stress kinase | Contig6447 | 1191 | Putative calcium-dependent protein kinase family protein | 36.88619447 | 33.32884215 | 33.3837441 | 0.813607541 |
| Stress kinase | Contig16500 | 1268 | CBL-interacting protein kinase 5 | 22.14137728 | 71.27940211 | 128.7084896 | 8.11E-20 |
| Stress kinase | First_Contig345 | 1237 | CBL-interacting protein kinase 25 | 52.66059554 | 68.54151637 | 150.587762 | 9.01E-13 |
| Stress kinase | Contig4430 | 1652 | CBL-interacting protein kinase 9 | 22.31329172 | 80.45709468 | 93.42027238 | 6.04E-12 |
| Stress kinase | Contig8200 | 716 | CBL-interacting protein kinase 22 | 69.11913104 | 127.9629756 | 136.3028262 | 1.31E-06 |
| Stress kinase | Contig9154 | 730 | CBL-interacting protein kinase 07 | 50.32734041 | 80.87985697 | 92.87695489 | 0.000308126 |
| Stress kinase | Contig8194 | 1837 | CBL-interacting serine/threonine-protein kinase, putative | 18.04175773 | 19.78050916 | 20.98812713 | 0.7529765 |
| Stress kinase | Contig9973 | 1295 | Serine/threonine-protein kinase SAPK3, putative | 75.38996299 | 118.287396 | 87.28710866 | 0.275492552 |
| Stress kinase | Contig1841 | 1586 | Serine/threonine-protein kinase SAPK6 | 49.88492444 | 54.36629051 | 56.7686823 | 0.440297444 |
| Stress kinase | Contig188 | 1571 | Putative snRK/SAPK family protein kinase | 52.10410172 | 57.37901658 | 70.39503232 | 0.087777228 |
| Defence/stress response | Contig12185 | 600 | Anamorsin homolog | 58.98394153 | 73.15328431 | 86.22585793 | 0.016099305 |
| Defence/stress response | Contig9406 | 1264 | DRE-binding protein DREB1 | 29.03330066 | 91.93496122 | 35.7307021 | 0.460499147 |
| Defence/stress response | Contig13202 | 576 | Cold-regulated LTCOR12 | 32.35262382 | 35.94926502 | 53.87848392 | 0.018390466 |
| Defence/stress response | Contig4018 | 943 | Glutathione peroxidase | 417.7220843 | 631.1557986 | 357.5947679 | 0.072611913 |
| Defence/stress response | Contig20945 | 492 | Putative WRKY DNA-binding domain superfamily protein | 35.88275607 | 39.40572981 | 54.84010277 | 0.036205627 |
| Defence/stress response | Contig2467 | 1245 | Actin7a | 891.1516896 | 919.0596748 | 842.3243803 | 0.516698343 |
| Defence/stress response | First_Contig53 | 673 | Golgi SNAP receptor complex member 1 | 57.01874798 | 40.39025433 | 48.28295777 | 0.498968755 |
| Defence/stress response | Contig17187 | 548 | Fad oxidoreductase, putative | 8.128550126 | 7.440503969 | 7.0622686 | 1 |
| Defence/stress response | Contig12014 | 998 | BZIP transcription factor ATB2 | 29.48282563 | 29.80060861 | 18.87722206 | 0.153971516 |
| Defence/stress response | Contig5309 | 1517 | GPAT | 46.28121241 | 37.99814704 | 41.17971657 | 0.750688405 |
| Defence/stress response | Contig1641 | 1099 | MYBR domain class transcription factor | 73.51502778 | 45.35774974 | 39.60020985 | 0.002584903 |
| Defence/stress response | Contig8110 | 1372 | Peptidyl-prolyl cis-trans isomerase | 142.7347921 | 119.4572423 | 181.9675339 | 0.017071351 |
| Defence/stress response | Contig23986 | 1121 | Copia LTR rider | 2.515420679 | 2.032600287 | 3.419814984 | 0.723273716 |
| Defence/stress response | Contig15029 | 626 | Elicitor-inducible LRR receptor-like protein EILP | 9.270030434 | 6.321841466 | 4.782538155 | 0.633775409 |
| Defence/stress response | Contig3033 | 731 | Arachidonic acid-induced DEA1 | 189.8529953 | 107.728742 | 133.0563997 | 4.64627E-05 |
| Defence/stress response | Contig23984 | 661 | Mitogen-activated protein kinase | 4.451416033 | 2.963311865 | 3.03794673 | 0.728306453 |
| Target protein compound | Contig13359 | 622 | 30S ribosomal protein S16-like | 14.97999302 | 13.30340155 | 15.55515753 | 0.858631261 |
| Target protein compound | Contig2441 | 957 | Chloroplast 30S ribosomal protein S10 | 58.54538493 | 42.06300273 | 35.7475743 | 0.030665075 |
| Target protein compound | Contig8973 | 455 | Putative alcohol dehydrogenase superfamily protein | 18.23273375 | 11.15770985 | 27.28264328 | 0.189442845 |
| Target protein compound | Contig10391 | 2304 | Class III homeobox-leucine zipper protein | 4.09728953 | 6.350111485 | 7.130975812 | 0.418503075 |
| Target protein compound | Contig25872 | 491 | Synaptotagmin-1 | 1.248466564 | 1.384044866 | 3.346184984 | 0.447449695 |
| Target protein compound | Contig18088 | 1431 | Core-2/I-branching beta-1,6-N-acetylglucosaminyltransferase | 5.568806483 | 6.285294387 | 7.169446206 | 0.603031568 |
| Target protein compound | Contig3753 | 1860 | Putative HAD superfamily hydrolase | 11.31517913 | 8.489202451 | 13.03389024 | 0.698076634 |
| Target protein compound | Contig11838 | 1149 | Class III homeobox-leucine zipper protein | 5.299481599 | 7.445201978 | 6.482299057 | 0.788106924 |
| Target protein compound | Contig1793 | 1621 | Heat shock cognate 70 kDa protein 2, putative, expressed | 313.6457622 | 240.9565508 | 694.6923611 | 6.73E-36 |
| Target protein compound | Contig4172 | 691 | CI small heat shock protein 2 | 288.667512 | 139.1296757 | 111.8037937 | 2.67E-18 |
| Target protein compound | Contig1500 | 463 | Heat shock protein 90-2 | 786.9664494 | 567.3266901 | 626.5943782 | 0.000156577 |
| Target protein compound | Contig17830 | 414 | Heat shock factor | 40.27420448 | 38.42956516 | 15.7859824 | 0.001325454 |
| Target protein compound | Contig3224 | 905 | Heat shock cognate 70 kDa protein, putative, expressed | 281.4593604 | 298.2846535 | 215.6747481 | 0.008122084 |

**Table S2 The analysis of variance (ANOVA) of different physiology measurements for *Lilium Lancifolium*.**

|  |  | **Sum of squares** | **Degree of freedom** | **Mean square** | **F value** | **P value** |
| --- | --- | --- | --- | --- | --- | --- |
| Soluble protein | Between groups | 0.0677 | 1 | 0.0677 | 6.8 | 0.007965 |
|  | Within groups | 19.8051 | 20 | 0.9903 |  |  |
|  | Total | 19.8727 | 21 |  |  |  |
| Starch | Between groups | 0.5013 | 1 | 0.5013 | 16.067 | 0.0007 |
|  | Within groups | 0.624 | 20 | 0.0312 |  |  |
|  | Total | 1.1253 | 21 |  |  |  |
| Soluble sugar | Between groups | 0.6256 | 1 | 0.6256 | 2.95 | 0.00593 |
|  | Within groups | 42.4186 | 20 | 2.1209 |  |  |
|  | Total | 43.0443 | 21 |  |  |  |
| MDA | Between groups | 1.7248 | 1 | 1.7248 | 21.218 | 0.0002 |
|  | Within groups | 1.6258 | 20 | 0.0813 |  |  |
|  | Total | 3.3506 | 21 |  |  |  |
